# Assessment of iodine status among pregnant women in a rural community in ghana - a cross sectional study

**DOI:** 10.1186/s13690-016-0119-y

**Published:** 2016-02-22

**Authors:** David L. Simpong, Patrick Adu, Rashid Bashiru, Martin T. Morna, Francis A. Yeboah, Kafui Akakpo, Richard K. D. Ephraim

**Affiliations:** Department of Laboratory Technology, School of Allied Health Science, University of Cape Coast, Cape Coast, Ghana; Institute of Infection and Inflammation, University of Glasgow, Glasgow, UK; School of Medical Sciences, University of Cape Coast, Cape Coast, Ghana; Ghana Energy Commission, Cape Coast, Ghana

**Keywords:** Iodine, Deficiency, Pregnant women, Ammonium persulfate

## Abstract

**Background:**

Pregnancy is associated with parallel increase in both iodine, and thyroid hormone requirements suggesting that, there may be the need for additional iodine intake during this period to prevent potential iodine insufficiency. Medically, an excess or reduced intake of this micronutrient has negative effects on the individual’s health. This study aimed at identifying the pattern of iodine levels among pregnant women at Kissi, Ghana.

**Method:**

A cross-sectional study was carried out among pregnant women on antenatal care at Kissi Health Centre (KHC) which serves the rural town with a population of about 4,500, located in the Komenda/Edina/Eguafo/Abirem (KEEA) municipality in the Central Region of Ghana. Demographic information, iodated salt usage and other pertinent information such as tobacco use were captured using a questionnaire. In addition, urine iodine concentration was estimated through the Ammonium per sulfate method after collecting on-the-spot urine samples.

**Results:**

Prevalence of iodine deficiency in pregnant women was 42.5 %. Of the 80 participants who were on iodized salt, only 16.25 % had mild iodine deficiency with none suffering from moderate or severe iodine deficiency. Of the 40 participants who did not use iodized salt, 35 %, 30 %, and 30 % suffered from severe, moderate and mild iodine deficiency respectively.

**Conclusion:**

The high prevalence of iodine deficiency reported in this study suggests that urgent national measures are required to correct iodine insufficiency in pregnant women in these communities.

## Background

The ongoing monitoring of population iodine status remains crucially important, and particular attention needs to be paid to vulnerable populations such as women of reproductive age, pregnant women, and younger children [1]. Iodine plays a vital role in the synthesis of thyroid hormone which subsequently exert effects on different organs and organ systems and is ultimately essential in the development of the central nervous system (CNS) during embryonic and foetal life [2]. This therefore suggests that iodine is an essential micronutrient during pregnancy. Not surprisingly, pregnancy is known to be associated with parallel increase in both iodine, and thyroid hormone requirements, possibly due to the physiological modifications emanating from the transfer of iodine and the thyroid hormone to the foetus [3]. Iodine requirements are increased from a value of 150 μg/day in adolescents and adulthood, to 250 μg/day in pregnancy [4]. Abnormalities of iodine metabolism are in two-folds: iodine excess or iodine deficiency. However, of the two abnormalities, iodine deficiency is more important [5]. This demonstrates the need for additional iodine intake [6] to prevent possible iodine insufficiency which is a concern during pregnancy.

The elementary pathology emanating from iodine deficiency had rested on endemic goiter [2], but studies in recent times have demonstrated a wide spectrum of disorders caused by iodine deficiency during pregnancy. These include stillbirth, increased number of spontaneous abortion, hearing defect in infants, congenital abnormalities, attention-deficit syndrome, irreversible mental retardation, impaired psychomotor development, and behavioral disorders [2, 4]. In spite of these adverse consequences, recent studies have demonstrated that iodine intake during gestation is low [7–9]. Consequently, the last decade has witnessed a substantial global progress in research relating to population iodine status, but information on iodine status among pregnant women in Ghana is scarce, possibly because routine screening and monitoring of this micronutrient in these individuals is lacking. Therefore, this study was aimed at identifying the pattern of iodine levels during pregnancy so as to generate data that would subsequently inform health policy makers about the effectiveness of the universal salt iodization (USI) program in Ghana.

## Methods

### Study site/study design

A cross-sectional study was conducted from December 2013 through to April 2014 among pregnant women resident in the Kissi township and receiving antenatal care at Kissi Health Centre (KHC). Kissi is a rural town located in Komenda/Edina/Eguafo/Abirem (KEEA) municipality in the Central Region of Ghana.

### Study population and ethical considerations

Ethical clearance was obtained from the Institutional Review Board of the University of Cape Coast (UCCIRB) and the authorities at KHC. Consented pregnant women (from first to third trimester) aged between 18-45 who were resident in the Kissi community were recruited for the study. Pregnant women with conditions or diseases that will affect urine iodine levels were excluded.

### Questionnaire

Questionnaires were administered to the participants to obtain demographic and obstetric data as well as information on iodated salt and tobacco (a substance known to affect iodine levels) use.

### Collection and determination of urine iodine levels

Participants were provided with clean containers into which they provided on-the-spot urine for the measurement of urine iodine concentration. UIC was estimated by the Ammonium persulfate method [10].

As a quality control measure, the interval between the time of addition of ceric Ammonium sulfate and the reading the absorbance were all the same for all samples, standards, and blanks so to rule out any systematic or random biases. In pregnant women, median urinary iodine levels of < 150 μg/L were considered as insufficient, 150–249 μg/L as adequate and >250 μg/L as more than adequate [11].

### Statistical analysis

All data were analysed using SPSS version 16 (IBM Corp.) and Minitab version 16 (Minitab Inc.). Data were analyzed using simple descriptive statistics such as frequency and percentages.

Age data showed a normal distribution with mean ≈ Median ≈ Mode and little variability. The mean and the standard deviation were used to measure the central tendencies and variability respectively. The ages were put into 6 categories with a class interval of 5. Simple descriptive statistics such as frequency and percentages were also used to analyze these categories.

Maternal Urine Iodine Concentration (UIC) during pregnancy was then determined in all analyses. The UIC data obtained was bimodal. It showed characteristics of skewness (positively skewed 1.01) with outliers (range, 5.2 μg/L – 1165.9 μg/L). The median served as the appropriate measure of central tendency for this data. The Median Absolute Deviation (MAD) was used to measure dispersion. Consequent to this, a nonparametric test, Mood Median test was used to assess the difference between demographic characteristics and the levels of the iodine concentrations with *p* <0.05 considered statistically significant.

## Results

A total of 120 pregnant women were involved in the study with an age range of ˃ 20 – ≤ 40 (mean age 27.20 ± 5.99 years). As shown in Table 1, only 12.5 % of the participants had sufficient iodine levels, with 45.0 % recording values above sufficiency. In addition, 42.5 % of the participant had insufficient UIC levels.

**Table 1:** Iodine levels among the pregnant women

Criteria	Urine iodine level (μg/L)	*n* = 120
Excessive	≥500	33 (27.50 %)
Above requirement	250–499	21 (17.50 %)
Iodine sufficient	150–249	15 (12.50 %)
Mild iodine deficiency	50–149	25 (20.83 %)
Moderate iodine deficiency	20–49	12 (10.00 %)
Severe iodine deficiency	<20	14 (11.67 %)

Table 2 shows the iodine levels in relation to the demographic characteristics of the pregnant women. Participants in the < 20 and 30–34 year categories had higher proportions with less than sufficient UIC levels i.e. 58.34 % and 54.17 % respectively. All participants in the ≥40- year group had less than sufficient UIC. In addition, participants in the 25–29-year category had the lowest proportion (34.28 %) having less than sufficient UIC. Moreover, participants in the 25–29-year category had the highest proportion (60 %) with UIC above sufficient levels. However, there was no significant difference between participant age group and their respective iodine level concentration (*p* = 0.272). Whereas a total of 50 % of participants in their first trimester had less than sufficient UIC, 39.3 % and 41.9 % of participants in their second and third trimester respectively, had less than sufficient UIC. 42.86 % of participants in their first trimester had above sufficient UIC, whereas 44.79 % and 32.25 % participants in their second and third trimesters had above sufficient UIC levels.

**Table 2:** Iodine levels in relation to the characteristics of the pregnant women

	Urine iodine levels *n* (%)						
*Age group (yrs)*	*<20*	*20–49*	*50–149*	*150–249*	*250–499*	*500*	*Total*
<20	2 (16.67)	2 (16.67)	3 (25.00)	1 (8.33)	2 (16.67)	2 (16.67)	12
20–24	4 (13.33)	2 (6.67)	5 (16.67)	4 (13.33)	9 (30.00)	6 (20.00)	30
25–29	2 (5.71)	2 (5.71)	8 (22.86)	2 (5.71)	7 (20.00)	14 (40.00)	35
30–34	3 (12.50)	3 (12.50)	7 (29.17)	6 (25.00)	1 (4.17)	4 (16.67)	24
35–39	2 (11.76)	3 (17.65)	1 (5.88)	2 (11.76)	2 (11.76)	7 (41.18)	17
≥40	1 (50.00)	0 (0.00)	1 (50.00)	0 (0.00)	0 (0.00)	0 (0.00)	2
*Occupation*							
Formal	0 (0)	0 (0)	1 (16.67)	1 (16.67)	1 (16.67)	3 (50)	6
Non-formal	11 (12.36)	9 (10.11)	19 (21.35)	11 (12.36)	15 (16.85)	24 (26.97)	89
None	3 (12.00)	3 (12.00)	5 (20.00)	3 (12.00)	5 (20.00)	6 (24.00)	25
*Trimester*							
First	2 (7.14)	4 (14.29)	8 (28.57)	2 (7.14)	5 (17.86)	7 (25.00)	28
Second	7 (11.48)	6 (9.84)	11 (18.03)	5 (8.20)	12 (19.97)	20 (32.79)	61
Third	5 (16.13)	2 (6.45)	6 (19.35)	8 (25.81)	4 (12.90)	6 (19.35)	31
*Use of iodated salt*							
Yes	0 (0.00)	0 (0.00)	13 (16.25)	14 (17.50)	20 (25.00)	33 (41.25)	0
No	14 (35.00)	12 (30.00)	12 (30.00)	1 (2.50)	1 (2.50)	0 (0.00)	0

Majority (95 %) of the participants had no formal occupation; and of these, 12.36 % had severe iodine deficiency, while 26.97 % had excess iodine levels. Although, fewer participants had formal occupation (5 %), none of the participants in this category had either severe or mild iodine deficiency. Of the 80 participants who were on iodized salt, only 16.25 % had mild iodine deficiency with none suffering from moderate or severe iodine deficiency. In contrast, of the 40 participants who did not used iodized salt, 35 %, 30 %, and 30 % suffered from severe, moderate and mild iodine deficiency respectively. In addition, 41.25 % of the participants on iodized salt had excess iodine levels whereas none of the participants who were not taking iodized salt had excess iodine levels.

## Discussion

Iodine is an important micronutrient required for the synthesis of thyroid hormones which is critical for proper development of the fetal brain. As foetal thyroid hormone production depends on maternal iodine levels [12], this study sought to investigate the UIC in pregnant women at Kissi the Central Region of Ghana.

The measurement of urinary iodine (UI) provides an accurate approximation of dietary iodine intake in view of the fact that the majority of iodine ingested is excreted via the urine. Urine iodine concentration is thus a reliable estimate of the amount of iodine intake and by extension of iodine deficiency [5]. This study found 42.5 % prevalence of iodine deficiency in pregnant women in the study population, suggesting that this at-risk group and their unborn children are still in danger of iodine deficiency disorder (IDD). Recent studies in the UK and Europe found that even a mild iodine deficiency during pregnancy led to defective neurodevelopment that negatively impacted the intelligence quotient (IQ) and cognitive functions of such children [13, 14]. Therefore, the high prevalence of iodine insufficiency reported in this study suggests that urgent national measures are required to correct the iodine insufficiency in pregnant women in these communities. The findings in this study also suggests that the high prevalence of iodine insufficiency may be due to non-compliance with the use of iodized salt. This is particularly so considering that none of the participants on iodized salt suffered from severe or moderate iodine deficiency, whereas a total of 65 % of the participants using non-iodized salt had either severe or moderate iodine deficiency. This is in line with the WHO/UNICEF Joint Committee on Health policy which recommended that universal salt iodization (USI) was the most cost-effective means to eradicate iodine insufficiency [15].

By using UIC in school-aged children (SAC), Andersson et al., previously found a 40 % prevalence of iodine insufficiency in an African population [1]. Although we used UIC in pregnant women in this study, the 42.5 % prevalence estimated in this study suggests a good correlation between the median UIC in SAC, and in adults, in a population that primarily uses iodized salt as the source of dietary iodine, as is the case in Ghana.

More importantly, this study in the Ghanaian setting also demonstrates non-compliance with the WHO universal salt iodization (USI) program proposed specifically to eliminate iodine deficiency disorders. Approximately, 33.3 % of the pregnant women evaluated were not using iodized salt stressing the need to find innovative ways to improve, as well as sustain, the educational programs meant to increase compliance with the WHO USI program. This would increase the understanding by the general public regarding the IDD and the need to use iodized salt to help eradicate these preventable diseases. In support of this argument, only a total of 2.5 % of participants who were not using iodized salt had UIC above sufficient levels. In contrast, a total of 66.3 % of the participants on iodized salt had UIC above sufficient levels. As it is documented that excess iodine is associated with adverse effects such as hyperthyroidism and thyroiditis [16], this study also demonstrates the need to routinely monitor the salt iodization program so as to ensure a median UIC of 100–200 μg/L as recommended by the WHO [17] in order to prevent iodine-induced hyperthyroidism and other adverse effects of excess iodine. Participants in the 25–29 year group had the lowest proportion of subjects with less than sufficient UIC as well as the highest proportion of subjects with more than sufficient UIC, suggesting that this age group may be more willing to take regular iodine supplementation in their diet.

To our knowledge this is the first study to use the persulfate method to estimate urine iodine in the Ghanaian population. However, this work has few limitations. First, a larger sample size would have shed more light on the correlation between participant age group and their compliance with the USI program. Second, although adequate amount of iodine (50 ppm) exist in iodized salt in Ghana, the methods of food processing, and cooking in individual households is likely to have affected the iodine levels in the participants. Third, this study is limited by our inability to obtain verification from independent bodies associated with quality controls of iodine assays. Finally, the iodine content in the local water supply was not determined.

## Conclusion

There is a high prevalence of iodine insufficiency in pregnant women visiting KHC in the Central Region of Ghana. Iodine insufficiency is more prevalent in pregnant women who do not use iodized salt as their main source of dietary salt. Urgent national policies including extensive educational programs to ensure compliance with the USI program, as well as fortification of foods with iodine will ensure adequate iodine levels especially in vulnerable individuals.
